# The Vital Force

**Published:** 1888-07

**Authors:** W. A. Hawley

**Affiliations:** Syracuse, N. Y.


### THE VITAL FORCE.

W. A. HAWLEY, M. D., SYRACUSE, N. Y.

*The cause of disease always dynamic.* An old, venerable, and venerated authority says, "The things of the spirit are discerned by the spirit." The life *is* the spirit, and it is not intellectually discerned nor demonstrated, but is its own proof and experience. It is the cause of all things in the animate world, and has power to raise inanimate matter up to its own plane. Its operation in living forms is called "The Vital Force." Now, while this is true, the vital force in one living form often is so entirely inimical to the vital force in another living form that if the two are brought in contact one is entirely driven out of its form, and the form is dead, has no life, no spirit. There are also in the inanimate world other forces, known as chemical forces, which, brought into contact with vital force in its various forms, will drive out of some of them the vital force, and so the form is dead. Now let it be noted that these things which we call vital force, chemical force, or even mechanical force, are each and every one *invisible, intangible, imponderable,* and immaterial. We apprehend none of them with our bodily eyes, we perceive none of them by our reason, but we apprehend them as we do our own life, by *experience,* or, as said above, they are discerned by the spirit. Moreover, these things are the causes of all things in nature, and therefore are the *real* things in nature of which nature is only the form. In this wonderful fact, this fundamental truth of truths, lies all the philosophy of life and death, of health and sickness. On it, here in this eleventh paragraph of the *Organon,* Hahnemann bases the whole superstructure of therapeutic science, the *science of healing.* If one is ever to become a real, an *enthusiastic* receiver of his system of medicine, he must get hold of this idea so that he *knows* that all *real* things are in the intangible realm—the realm of *Forces.*

Further then, forces being the *cause* of all forms, they must exist before the forms, and therefore *may, yes, must,* exist after them. When this is seen the cause of disease and the mode by which it may be cured becomes entirely intelligible and all the difficulties concerning the much disputed matter of potentization vanish. For it is always the invisible force residing in the form and not the form itself which is the cause of any phenomenon. Your watch is stopped. Examination reveals a particle of sand has somehow found its way to a lodgment between the cogs of two connecting wheels and the watch is stopped. What stopped it? The sand, surely. Was it the form that did it? Take out that intangible, invisible something called chemical affinity and instantly it vanishes into its original elements and your watch is again in motion. Thus we see that even in this mechanical disturbance the cause is dynamic. In this paragraph of the *Organon* the author asserts that the cause of every departure from health is some such intangible force, recognized only by the symptoms which it produces. This we can see to be true in an illness caused by a fright or violent anger, but in a case of poisoning, by a snake bite, for instance, what is it? Evidently not the material of the virus, as is evinced by the rapidity of its transmission over the whole man, but it is that peculiar something, *intangible but real,* which makes the *snake,* is indeed the *real snake,* and of which the form of the virus is the vehicle. The materialist may tell you that it is the action of the substance of the virus on the cell which causes the result, presumably by a combination of the substance with the substance of the cell. Grant it. What causes the combination? That is just the question which is only answered by the existence of an intangible, invisible, immaterial something which we call force or dynamics.

But says the materialistic scientist, "Force is only a quality of matter. I put my hand on this table and I find there is force there." Yes, who says no? Still, force is not matter, but that which aggregates matter. Some one has well said, "Force is order, that which renders chaos cosmos." What is the universe but an infinity of forces harmonious ever? The cause of all effects, it makes every effect the cause of some other effect. Yet we cannot see it and only know it as we experience it, and while experiencing it continually, we are scarcely conscious of it, unless it be something which disturbs the harmony of the forces which we ourselves *are*. The application of this subject to the cure of disease need not here be considered. We shall come to that later.

